# Pulmonary blood density in systemic sclerosis – a novel non-invasive measure of pulmonary arterial hypertension?

**DOI:** 10.1186/1532-429X-11-S1-P183

**Published:** 2009-01-28

**Authors:** Mikael Kanski, Håkan Arheden, Roger Hesselstrand, Martin Ugander

**Affiliations:** 1 Department for Clinical Physiology, Lund, Sweden; 2 Department for Rheumatology, Lund, Sweden

**Keywords:** Magnetic Resonance Imaging, Pulmonary Arterial Hypertension, Pulmonary Vein, Left Atrium, Lung Volume

## Introduction

Patients suffering from systemic sclerosis (SSc) have a highly increased risk of developing pulmonary arterial hypertension (PAH). An early detection of PAH is important in order to halt the progress of disease. Currently, the assessment of pulmonary pressures lacks reliable quantitative non-invasive measures.

## Purpose

The aim of this study was therefore to use magnetic resonance imaging (MRI) to measure the changes in pulmonary blood volume (PBV), the PBV variation (PBVV) throughout the cardiac cycle, and the pulmonary blood density (PBD) in early-stage SSc and to make a preliminary comparison to healthy individuals.

## Methods

Thirty-nine SSc patients (25 women and 14 men, 31–81 years, mean 58 years) and healthy patients and healthy volunteers (three women and 10 men, 21–45 years, mean 26) underwent cardiac MRI. PBV was calculated as the product of cardiac output determined by velocity encoded MRI, and the pulmonary transit time (PTT) determined as the time for a 2 ml intravenously administered contrast bolus to pass from the pulmonary trunk to the left atrium. The lung volume was determined by planimetry using transversal MR images covering the lungs. The PBD was defined as the PBV divided by the lung volume. Also, the blood flow in the pulmonary artery and the pulmonary veins was measured using velocity encoded MRI. The PBVV was calculated by integration of the difference in arterial and venous pulmonary flow over the cardiac. PBV and PBD was measured in three patients (all men, 24, 34, and 45 years, respectively) who showed to be healthy. In 10 healthy volunteers (three women and 7 men, 21–30 years, mean 24 years), the PBVV was assessed. The SSc patients were then compared to the healthy subjects considering PBV and PBD, and PBVV, respectively.

## Results

Stroke volume, PBV and PBD in 36 SSc patients and three healthy subjects were respectively (mean ± SD) 77 ± 20 ml vs 94 ± 11 ml, 467 ± 111 ml vs 552 ± 99 ml, and 17 ± 5% vs 19 ± 6%. PBVV in 32 SSc patients and 10 healthy individuals were respectively 31 ± 9 ml vs 45 ± 14 ml.

## Conclusion

This study is the first to show the feasibility to assess the PBD using MRI. Preliminary data shows no difference between newly diagnosed SSc patients and healthy individuals considering PBD. The PBD may be a useful prognostic non-invasive measure of PAH and cardiac failure. Therefore, further studies are needed to reveal the importance of the PBD in these contexts.

